# A111 A RECTUM-SPECIFIC SELECTIVE RESECTION ALGORITHM OPTIMIZES ONCOLOGIC OUTCOMES FOR LARGE NON-PEDUNCULATED RECTAL POLYPS

**DOI:** 10.1093/jcag/gwab049.110

**Published:** 2022-02-21

**Authors:** N C Shahidi, S Vosko, S Gupta, A Whitfield, O Cronin, T O’Sullivan, W van Hattem, M Sidhu, D Tate, E Lee, N Burgess, S Williams, M Bourke

**Affiliations:** 1 Westmead Hospital, Department of Gastroenterology and Hepatology, Sydney, New South Wales, Australia; 2 University Hospital of Ghent, Ghent, Belgium

## Abstract

**Background:**

Endoscopic mucosal resection (EMR) and endoscopic submucosal dissection (ESD) are complementary techniques for large (≥ 20mm) non-pedunculated rectal polyps (LNPRPs). A mechanism for appropriate technique selection has not been described.

**Aims:**

To evaluate whether a selective resection algorithm using EMR and ESD, based on real-time optical evaluation, optimizes oncologic outcomes for LNPRPs

**Methods:**

We evaluated the performance of a selective resection algorithm (SRA; 08/2017-04/2021) compared to a universal EMR algorithm (UEA; 07/2008-07/2017) for LNPRPs within a prospective observational study. In the SRA, LNPRPs with features of superficial submucosal invasive cancer (SMIC < 1000μm; S-SMIC; Kudo pit pattern Vi), or with an increased risk of SMIC (Paris 0-Is or 0-IIa+Is non-granular, 0-IIa+Is granular with a dominant nodule ≥ 10mm) underwent ESD. The remaining LNPRPs underwent EMR. Algorithm performance was evaluated by SMIC identified after EMR, curative oncologic resection (R0 resection, S-SMIC, absence of negative histologic features), technical success, adverse events, and recurrence at first surveillance colonoscopy.

**Results:**

480 LNPRPs were evaluated (290 UEA, 190 SRA). Median lesion size was 40mm (IQR 30-60mm). In the SRA, 103 (54.2%) and 87 (45.8%) LNPRPs underwent EMR and ESD, respectively. SMIC was identified in 56 (11.7%) LNPRPs. Significant differences in SMIC after EMR (SRA 1 (1.0%) vs. UEA 35 (12.1%); p = 0.001), curative oncologic resection (SRA 7 (33.3%) vs. UEA 2 (5.7%); p = 0.010), and recurrence (SRA 2 (1.6%) vs. UEA 40 (17.2%); p < 0.001) were identified. No significant differences in technical success or adverse events were identified (all p > 0.137). Among potentially curable malignant LNPRPs which underwent ESD, 100% (7/7) were cured.

**Conclusions:**

A SRA optimizes oncologic outcomes for LNPRPs and mitigates the risk of piecemeal resection of cancers.

**Funding Agencies:**

The Cancer Institute of New South Wales provided funding for a research nurse and data manager to assist with the administration of the study. Neal Shahidi was supported by the University of British Columbia Clinician Investigator Program. There was no influence from either institution regarding study design or conduct, data collection, management, analysis, interpretation, preparation, review, or approval of the manuscript.

**Table:** Oncologic Outcomes for Malignant LNPRPs

	Overall LNPRPs (N = 56, %)	UEA LNPRPs (N = 35, %)	SRA LNPRPs (N = 21, %)	P-value
SMIC after EMR*	36 (9.2)	35 (12.1)	1 (1.0)	0.001
En Bloc Resection	23 (41.1)	4 (11.4)	19 (90.5)	<0.001
R0 Resection	20 (35.7)	2 (5.7)	18 (85.7)	<0.001
Curative Resection	9 (16.1)	2 (5.7)	7 (33.3)	0.010

EMR, endoscopic mucosal resection; LNPRP, large non-pedunculated rectal polyp; SMIC, submucosal invasive cancer; SRA, selective resection algorithm; UEA, universal endoscopic mucosal resection algorithm *Denominator: Large non-pedunculated rectal polyps which underwent endoscopic mucosal resection

